# Early warning scores and critical care transfer – patient heterogeneity, low sensitivity, high mortality

**DOI:** 10.1007/s11845-021-02558-7

**Published:** 2021-03-10

**Authors:** Claire C. Nestor, Maria Donnelly, Siobhán Connors, Patricia Morrison, John Boylan

**Affiliations:** 1 Tallaght University Hospital, Dublin, Ireland; 2 St.Vincent’s University Hospital, Dublin, Ireland; 3 University of Hong Kong, Department of Anaesthesiology, Hong Kong

**Keywords:** Critical care, Early warning, Intensive care unit, Mortality, Observational study, Screening

## Abstract

**Background:**

Emergency warning systems (EWS) are becoming a standard of care, but have unproven screening value in early critical illness. Similarly, emergency response team (ERT) care is of uncertain value. These questions are most controversial in mixed patient populations, where screening performance might vary, and intensivist-led ERT care might divert resources from existing patients.

**Aims:**

To examine triggering events, disposition and outcome data for an intensivist-staffed EWS-ERT system.

**Methods:**

We analysed process and outcome data over three years, classing EWS-triggered patients into three categories (non-escalated, escalated ward care and critical care transfer). The relationships between EWS data, pre-triggering clinical data, and patient disposition and outcome were examined.

**Results:**

There were 1675 calls in 1190 patients. Most occurred later during admission, with critical care transfer in a minority; the rest were followed by escalated or non-escalated ward care. Patients transferred to critical care had high mortality (40.3%); less than half of patient transfers occurred following triggering EWS score predicted overall hospital mortality, but not mortality after critical care.

**Conclusions:**

In a diverse hospital population, most triggering patients did not receive critical care and most critical care transfers occurred without triggering. Triggering was an insensitive screening measure for critical illness, followed by poor outcome. Higher scores predicted higher probability of transfer, but not later mortality, suggesting that EWS is being used as a decision aid but is not a true severity of illness score. Other, non-EWS data are needed for earlier detection and for prioritizing access to critical care.

Emergency warning score (EWS) systems are a simple, widely used screening method for imminent patient deterioration, and are becoming a standard of acute care [1]. Though not validated in the detection of early critical illness and lacking proven impact on outcome, EWS data are often used as a triage tool, triggering calls to emergency response teams (ERTs). Relative to standard care, the effect of ERT-delivered care on outcome is also uncertain, with no randomised data supporting its use [2, 3]. Despite these limitations, and a lack of consensus on optimum staffing models, these teams have been strongly recommended as a part of inpatient safety [4].

Difficulties in linking EWS triggering to better outcome may relate to patient heterogeneity [5]. EWS sensitivity and specificity may vary in different patient sub-populations. In some subgroups, abnormal vital signs might have low sensitivity for organ dysfunction, and even bias clinicians towards later detection of critical illness [6]. Triggering thresholds may discriminate poorly (low specificity) between patients who need critical care and those with non-critical illness. Limited validation of EWS data in decision-making might lead to mis-triaging, e.g., critical care transfer of patients with non-critical conditions. As a result, routine clinical use of these systems without robust evidence might address an unmet need, but risk mis-allocation of critical care resources to patients not in need. Also, the unvalidated use of trigger-based criteria in critical care research might undermine study design, as inclusion of patients without critical illness would reduce study power. For these reasons, it is important to understand the contribution of EWS/ERT-based care to critical care decision-making and its association with outcome.

We used data collected following the inception of an intensivist-staffed university hospital ERT system to examine triggering events, disposition and outcomes in a diverse general population. The study examined process of care and outcomes in ward inpatients to assess treatment escalation, critical care transfer and post-critical care mortality.

## Methods

The study was a retrospective analysis of prospectively collected data from the ERT and critical care databases at Tallaght University Hospital, a university-affiliated tertiary referral hospital, with a single nine-bedded general ICU, and variable surge capacity which included coronary care, high-dependency, PACU beds and transfer to an unit ‘off site’. ICU occupancy was 103%, 95% and 99% for 2013/2014/2015, respectively, with respective SMRs of 0.6, 0.7 and 0.6 [7–9]. As patient anonymity was preserved, the need for consent was waived by the local institutional review board.

## Data source & collection

1. The study population consisted of 1190 ward inpatients, including a small group of patients (n = 96) with DNAR orders already in place, who received ERT care following a valid triggering event during the period 1 January 2013—31 December 2015. The hospital uses the NEWS (National Early Warning Score) system for event detection, as mandated by the national Department of Health shown in Table 1 [10]. The algorithm instructs nursing staff to activate the system in the presence of a total score of 7 or greater, a score of 3 in a single variable, or any concern about a patient’s condition. Calls generated for events not meeting these criteria were excluded from analysis. Care was provided by an unfunded, non-dedicated ICU team, i.e., staff resources were diverted to provide ward patient assessment and treatment. All data were recorded, verified and retrieved by a dedicated nurse coordinator using the EWS database, and the ICU database (IntelliVue Clinical Information Portfolio, Koninklijke Philips Electronics, 2010). Hospital admission, length of stay and mortality data were obtained from the Inpatient Information Management System and the Hospital Inpatient Enquiry System. Quarterly compliance checks with the use of the NEWS observation chart showed compliance rates of > 85% during 2013–2015. Data points routinely collected are shown in Table 2.

**Table 1:** VitalPAC™ Early Warning Score (ViEWS) variables of interest and score key

Variable	3	2	1	0	1	2	3
Respiration rate	≤ 8		9–11	12–20		21–24	≥ 25
Sp0_2_ (%)	< 91	92–93	94–95				
Inspired Oxygen				Air			Any oxygen
Temperature (°C)	≤ 35.0		35.1–36	36.1–38	38.1–39	≥ 39.1	
Systolic Blood Pressure (mmHg)	≤ 90	91–100	101–110	111–249	≥ 250		
Heart Rate (BPM)		≤ 40	41–50	51–90	91–110	111–130	≥ 131
AVPU/CNS response				Alert(A)			Voice(V)/ Pain(P)/ Unresponsive(U)

Physiological variables and scoring key for the ViEWS system

**Table 2:** Variables of Interest

Patient Demographics, EWS-related data	Disposition, Outcomes
Age	Disposition
Sex	Remained on ward
Hospital admit date	Care escalated
Date and time of call	Care limited
ViEWS score	Transferred (ICU, PACU, HDU, theatre, off site)
Parameter scores	
Retrigger	Outcomes
Time spent by ERT staff at call	Died after ward care
Investigations ordered	Died after critical care
Interventions performed	

Variables of interest: Patient demographics and call data, disposition and outcome

Each triggering event was followed by a consultation between the team physician and the referring team, and a decision was made to either escalate or limit care, in accordance with hospital protocol. In patients receiving escalated care, disposition options were to receive an increased level of (escalated) ward care or to be transferred to a unit providing a higher level of care, as decided by discussion between the team physician and an intensive care specialist.

Variables of interest are shown in Table 2. The primary study endpoints were disposition and outcome-related, while the secondary endpoints related to process of care (ward investigations, interventions and time spent by nurse and doctor at the call). The three primary endpoints were: (1) a decision to escalate care (either at ward or critical care level), (2) in patients receiving escalated care, a decision to provide critical care vs. ward care, and (3) total in-hospital mortality in patients receiving critical care. Clinical and demographic variables included patient gender, age and medical/surgical status, duration of hospital stay before triggering, EWS score, time of call (in-hours vs. out of hours), number of triggering events, and level of intervention. Out of hours calls were defined as calls taking place outside the weekday working hours of 9 AM to 5 PM. Before analysis, we took data from all non-DNAR patients, ranked EWS scores in relation to in-hospital mortality, and regraded them as ordinal data on a 16-point scale. For disposition and outcome analyses, the model used clinical and EWS data from patients’ final triggering event.

To estimate the sensitivity of ERT as a screening methodology for ward critical illness, we recorded the number of non-ERT ward transfers to critical care during the study period.

## Statistical analysis

Data were indexed per 1000 hospital admissions where appropriate. Process of care data are presented in relation to calls, and therefore include multiple calls in a minority patients. We analysed event frequencies over time using chi-squared or the chi-squared test for trend, as appropriate. Within-group frequencies were evaluated using chi-squared or the exact probability test, as appropriate. Univariate outcome prediction was carried out using Spearman’s ρ and Kruskal–Wallis testing. We used stepwise logistic regression analysis to identify independent predictors of escalated care, critical care transfer and post-critical care mortality. Statistical analyses were performed using Stata 12 (Statacorp, College Station, TX).

## Results

### Call activity, investigations and interventions

**Fig. 1:**
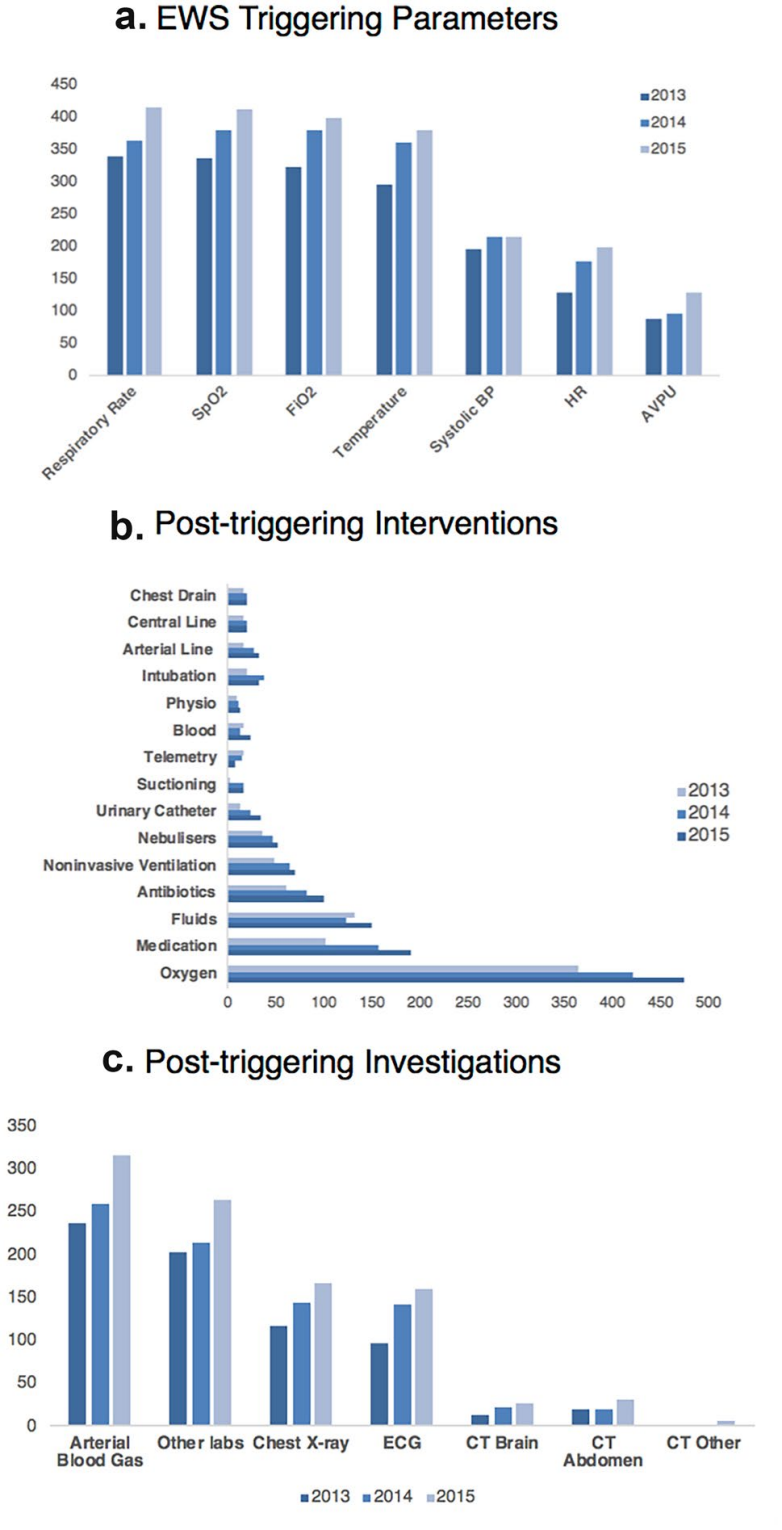
EWS Triggering Parameters, Post-ERT Interventions and Post-ERT Investigations

Call activity and demographics are displayed in Table 3. During the study period there were 1675 calls in 1190 patients; in 111 calls (96 patients) a DNAR order was in place at the time the system was triggered. A total of 54,787 hospital admissions took place during this time (call/admission ratio of 0.03:1). Other than for raw mortality, data on disposition and outcomes are omitted for DNAR patients. The proportion of triggering events in relation to admissions increased over the study period, attributable to an increased call rate in medical patients. Other demographic and call patterns were stable over time, with no seasonal variation (data not shown). Triggering data are summarised in Fig. 1a. The commonest abnormalities were altered respiratory rate, oxygen saturation and inspired oxygen requirement. Apart from a difference in the number of respiratory events (P < 0.03), EWS abnormalities and scores were similar from year to year. Most triggering events occurred later during admission, with a minority of calls during the first 72 h.

**Table 3:** Call Demographics

Year	2013	2014	2015	P
Total calls	486	578	611	
Calls/1000 admissions	26	32	34	0.0001
Age	71 (59, 80)	72 (60, 81)	71 (61, 81)	> 0.10
Male	183 (49)	234 (53)	260 (56)	> 0.10
Medical	299	318	418	0.001
Surgical	187	260	193	> 0.10
Working hours	125 (26)	129 (22)	149 (24)	> 0.10
Out of hours	361 (74)	449 (78)	462 (76)	> 0.10
Single trigger, critical care	72 (24)	92 (27)	82 (22)	0.40
Multiple trigger, critical care	34 (46)	45 (47)	41 (43)	0.40
Calls (1/2 +)	302 / 184	346 / 232	369 / 242	0.74
Timing of call (day 1 / 2 / 3 / later)	99 / 45 / 49 / 293	124 / 49 / 52 / 353	130 / 69 / 43 / 369	0.44

Call numbers and demographics. Data are absolute values, medians (IQR) or numbers (percentages)

Interventions and investigations are described in Figs. 1b, c, respectively. Most procedures were non-invasive (oxygen, antibiotics, etc.). Other than increasing procedure frequency, other demographics and call patterns were stable over time, with no yearly or seasonal variation (data not shown).

## Patient disposition and outcomes

A minority of calls (388/1675, 23.2%) resulted in critical care transfer following assessment. Most calls (1287/1675, 76.8%) were followed by either escalated or non-escalated ward care; critical care transfer rates did not change during the study period (data not shown). Data for final disposition and outcome (n = 1190) (escalated vs. non-escalated care, critical care vs. escalated ward care) and post-critical care mortality are presented in Table 4. Multivariate predictors of escalated care, critical care transfer and post-critical care mortality are presented in Table 5.

**Table 4:** Ward Care, Critical Care and Post-Critical Care Mortality

A – Escalated vs. Non-escalated Ward Care			
	ESCALATED	NON-ESCALATED	P
	(n = 879)	(n = 311)	
Age	69 (56, 79)	79 (69, 86)	< 0.001
Female	410 (47)	160 (51)	0.15
Medical	550 (63)	261 (84)	< 0.001
EWS score	8 (6, 9)	8 (7, 10)	< 0.01
Pre-EWS length of stay (days)	4 (1, 13)	9 (2, 24)	< 0.001
Out of hours event	645 (73)	246 (79)	< 0.05
Multiple triggering	169 (19)	77 (25)	< 0.05
Noninvasive ventilation	98 (11)	41 (13)	0.34
Tracheal intubation	68 (8)	7 (2)	< 0.01
B – Critical Care vs. Escalated Ward Care			
	CRITICAL CARE	WARD CARE	P
	(n = 310)	(n = 569)	
Age	69 (58, 78)	69 (54, 79)	0.66
Female	141 (45)	269 (47)	0.61
Medical	196 (63)	354 (62)	0.77
EWS score	8 (7, 10)	7 (5,9)	< 0.01
Pre-EWS length of stay (days)	5 (1, 15)	4 (1, 11)	0.08
Out of hours	226 (73)	419 (74)	0.81
Multiple triggering	87 (28)	82 (14)	< 0.01
Noninvasive ventilation	61 (20)	37 (7)	< 0.01
Tracheal intubation	59 (19)	9 (2)	< 0.01
C – Post-Critical Care Mortality vs. Survival			
	DIED	SURVIVED	P
	(n = 125)	(n = 185)	
Age	74 (66, 82)	64 (54, 73)	< 0.01
Female	59 (47)	82 (44)	0.62
Medical	92 (74)	104 (56)	< 0.01
EWS score	8 (7, 10)	8 (7, 10)	0.72
Pre-EWS length of stay (days)	5 (2, 18)	4 (1, 13)	0.04
Out of hours	90 (72)	136 (74)	0.81
Multiple triggering	38 (30)	49 (26)	0.45
Noninvasive ventilation	24 (19)	37 (20)	0.86
Tracheal intubation	28 (22)	31 (17)	0.21

Demographic and clinical data for disposition (escalated vs. non-escalated ward care (A), escalated ward vs. critical care (B), and outcome (post-critical care mortality vs. survival (C)).

Data are numbers (%) or medians (IQR)

**Table 5:** Multivariate Model of Escalation, Critical Care Transfer and Post-Critical Care Survival

		A. Escalation		B. Critical Care		C. Death	
	OR (95% CI)		P	OR (95% CI)	P	OR (95% CI)	P
Age	0.95 (0.94–0.96)		< 0.01			1.07 (1.05–1.09)	< 0.01
Medical vs. Surgical	0.30 (0.20–0.46)		< 0.01			3.11 (1.78–5.44)	< 0.01
Days pre-EWS	0.991 (0.986–0.996)		< 0.01			1.010 (1.005–1.020)	0.03
Intubation	5.82 (2.02–16.75)		< 0.01	19.6 (8.8–43.3)	< 0.01		
NIV				3.4 (2.1–5.6)	< 0.01		
EWS grade	0.91 (0.85–0.98)		0.02	1.2 (1.1–1.3)	< 0.01		
Multiple triggers				2.3 (1.6–3.3)	< 0.01		
AVPU				0.52 (0.36–0.77)	< 0.01		
BP				1.54 (1.11–2.15)	0.011		
Temperature	1.97 (1.21–3.21)		< 0.01	0.65 (0.43–0.97)	0.036		

Multivariate predictors of patients receiving escalated vs. non-escalated ward care (A), patients receiving critical care vs. escalated ward care (B), and post-critical care mortality (C).

Relative to those given non-escalated care, patients receiving escalated care were younger, more likely to have been admitted surgically, and had been in hospital for a shorter time. They were also more likely to have had a single triggering event, to have deteriorated during normal working hours, to have lower scores, and to have needed tracheal intubation. Individual score components associated with escalation were desaturation and abnormal body temperature. The only multivariate predictors of escalated vs. non-escalated care were lower patient age, surgical status, shorter duration of stay before triggering, tracheal intubation, lower triggering score and body temperature. The multivariate model was moderately successful in discriminating escalation from non-escalation (C statistic = 0.76, P < 0.001).

Patients transferred to critical care were more likely to need either invasive or noninvasive respiratory support and have had multiple triggering events. They also had higher global scores and greater abnormalities of most individual score elements (respiratory rate, oxygen saturation, FiO_2_ requirement, heart rate and blood pressure); abnormal AVPU scores were more frequent in patients receiving escalated ward care. The only multivariate predictors of critical care transfer were: tracheal intubation or noninvasive ventilation, higher scores and blood pressure abnormalities, and multiple triggering events. AVPU and body temperature abnormalities were independent predictors of escalated ward care rather than critical care. The multivariate model had weak discriminative performance (C statistic 0.68, P < 0.01).

## Mortality

Including those who had already DNAR status, a total of 405 patients died after triggering (34.0%). After excluding DNAR patients, there was a monotonic relationship between hospital mortality and score for the study population as a whole (ρ = 0.17, P < 0.0001). Hospital mortality was extremely high in patients in whom treatment limitation was put in place after triggering (189 of 215 patients, (87.9%)) and was also high in patients transferred to critical care after triggering (125 of 310 patients, (40.3%)). In ward patients with escalated care, mortality was similar after both single and multiple triggering events (16/487 patients vs. 4/83 patients, respectively, P = 0.48).

Univariate and multivariate predictors of mortality after critical care transfer are presented in Table 4 C and Table 5, respectively. In patients receiving critical care there was no relationship between in-hospital mortality and either total score or individual score elements. Gender, out of hours triggering, multiple triggering episodes, and need for ventilatory support were not associated with mortality. The sole univariate and multivariate predictors of death after critical care were increased age, medical status and longer duration of inpatient stay before the triggering event. The multivariate model was moderately successful in discriminating in-hospital death after critical care (C statistic = 0.76, P < 0.001).

## Discussion

In a model of triage activity of intensivist-led emergency response team care at the bedside soon after EWS triggering, we found that the system detected a minority of critically ill patients with unusually high post-transfer mortality, as well as large subgroups of patients with subcritical and pre-terminal illness who received escalated ward care and limitation of treatment goals, respectively. Most critical care transfers took place late in the hospital stay, and triggering had low sensitivity for transfer. Higher scores predicted higher probability of transfer, but not later mortality. Although EWS score may be used in triaging decisions, it is not a true severity of critical illness score, and has limited usefulness in prioritizing care. The only predictors of inpatient mortality in EWS-transferred patients were age, medical status and longer prior duration of hospital stay.

In the current study, many triggering events were followed by a re-evaluation of treatment goals, and patients with treatment limitation had a predictably low survival rate (less than 15%). Despite similar median scores, the overall distribution tended to be higher in ward patients in whom treatment was not escalated. Triggering may have played a role in decisions to limit care, but the retrospective design of the study means that this cannot be quantified. Intensivist-provided emergency care typically aims to detect and treat patients with treatable serious illness, though other authors have proposed an extended role for intensivists in the care of patients with serious comorbidities in whom the decision to provide critical care may be controversial [11]. We would argue that a combination of resource constraints, low yield from EWS data and a high proportion of non-critical illness indicate a need for re-evaluation of the role of intensive care physicians in the first responder tier.

**Fig. 2:**
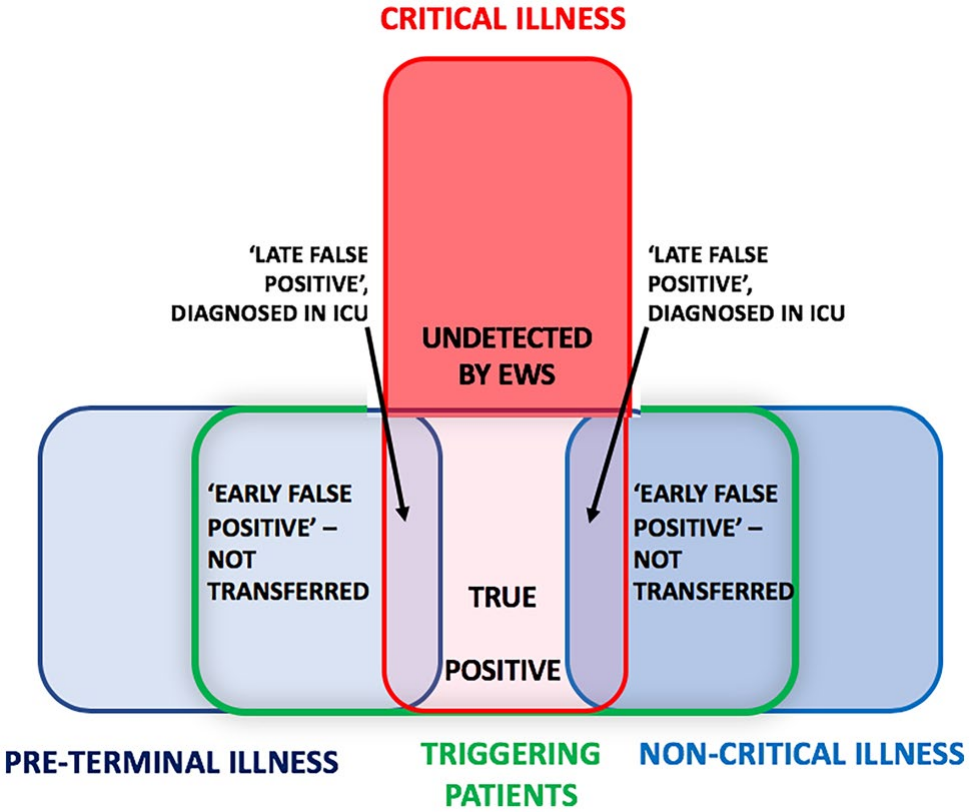
Performance of EWS

Most triggering events resulted in non-critical care interventions. Less than a quarter of triggering patients were ultimately transferred to critical care, with a high mortality rate. There are multiple possible explanations for this. First, there may have been selection bias: patients with ‘triggering’ critical illness may have had more severe illness than ward patients transferred by alternative pathways [12]. Second, high ICU occupancy may have curtailed access, with less severely ill patients receiving escalated ward care and surviving who might still have benefited (e.g., reduced morbidity) from critical care. Third, some patients with undiagnosed pre-terminal illness may have received critical care after a triggering event, only for futility to be recognized later. Fourth, ICU ‘strain’ – a mismatch between demand for care and the unit’s ability to provide it might have been increased by ERT activity, worsening outcome in some patients [13]. The performance of EWS in the study is described schematically in Fig. 2, showing both low sensitivity and two variants of ‘false positive’ triggering: early ‘false positives’, resolved at ERT/ward level, and late ‘false positives’, which are diagnosed after critical care transfer. Our data suggest that outreach planning should include estimates of the prevalence of undetected early critical illness and its possible impact on unit strain.

The widespread use of EWS scores is based on their clinical and prognostic relevance. A well-documented relationship between abnormal ward vital signs and all-cause hospital mortality gives these data face validity as a screening method for early critical illness, even though their role in these patients is uncertain, and randomized trial data inconclusive [14]. In the current study, scores predicted overall hospital mortality, but had no prognostic relationship in patients receiving critical care. In heterogeneous unstratified populations, estimates of the relationship between EWS abnormalities and patient outcome are likely to be biased, with the abnormalities seen in the highest mortality subgroup dominating the analysis. Here, triggering data had a variable relationship with patient disposition; higher scores were followed by non-escalation of ward care in one subgroup, and predicted critical care transfer in another. This disparity has implications for study design. If many patients with early critical illness fail to trigger the system, and triggering patients with critical illness have unusually high mortality, this limits the utility of EWS scoring as an entry criterion for clinical research studies.

Introduction of emergency response team-based care has been linked to a lower incidence of cardiac arrest and reduced hospital mortality, evidence characterized as ‘moderately strong’ [15–19]. If simple vital signs performed well in detecting and risk stratifying ward patients with early critical illness, this would be a persuasive reason for their use as a screening method, even without trial-based evidence for benefit. Counterarguments include: (1) in heterogeneous hospital populations, triggering might occur in different clinical contexts, many without underlying critical illness, or might not occur at all, and (2) triggering might occur late rather than early, with intervention after organ dysfunction has progressed. We found evidence for both of these arguments; in particular, many ward patients were being transferred to critical care without triggering, implying a sensitivity lower than that considered acceptable for a screening technique. Also, despite predicting critical care transfer and overall hospital mortality, EWS data failed to predict mortality in those receiving critical care. With only three positive predictors in a model with many events, this is unlikely to be related to inadequate study power. Despite a similarity to physiology-based scoring systems such as APACHE and SOFA, EWS data are probably not a true severity of critical illness score. Prognostic models for general in-hospital mortality suggest that most predictive power stems from demographic and early post-admission laboratory data [20], suggesting a dominant role for early events in many seriously ill patients. Meanwhile, the high percentage of patients transferred without triggering suggests that monitoring of vital signs is an ineffective screening modality for early critical illness. Further research efforts in non-triggering patients may yield insights into the limitations of EWS-based screening.

Our study has several limitations. First, its single-centre design raises the issue of generalizability. However, we suspected that even with the low practice variation occurring at one institution, an EWS system might perform differently with varying casemix, and our data confirm that in a heterogeneous patient population, systems based on vital signs alone have very limited capacity to capture early, treatable critical illness. Second, the validity of critical care transfer as a reference standard depends on all transferred patients having treatable critical illness. Without formal adjudication, the true incidence of treatable critical illness cannot be determined. Binary decisions (transfer vs. no transfer) are based on non-binary judgments (treatable critical illness ‘present’ vs. ‘absent’ vs. ‘uncertain’) [21], making some patients likely to receive critical care for non-critical or pre-terminal illness. This ‘late false positive’ subgroup further reduces the positive predictive value of EWS-based screening, without affecting its low sensitivity.

In conclusion, we found that EWS ward triggering in a mixed general hospital setting identified a population with a mortality risk far in excess of ward baseline values. Triggering occurred late in patients’ stay, detected a subgroup of seriously ill patients with high mortality, and accounted for less than half of ward transfers to critical care. The screening performance of EWS data is undermined by the more frequent use of other criteria for critical care transfer, suggesting that its value is casemix-dependent and might vary between – or even within – institutions. EWS scores may be used in patient triage, but are not independently predictive of post-critical care mortality, suggesting that other data should be used at the bedside in prioritising access to critical care.

## Data Availability

The data that support the findings of this study are available from the corresponding author, CC Nestor, upon reasonable request.
