# Correction to: Early warning scores and critical care transfer – patient heterogeneity, low sensitivity, high mortality

**DOI:** 10.1007/s11845-021-02616-0

**Published:** 2021-04-12

**Authors:** Claire C. Nestor, Maria Donnelly, Siobhán Connors, Patricia Morrison, John Boylan

**Affiliations:** 1 Tallaght University Hospital, Dublin, Ireland; 2 St.Vincent’s University Hospital, Dublin, Ireland; 3 Department of Anaesthesiology, University of Hong Kong, Pok Fu Lam, Hong Kong

## Correction to: Irish journal of medical science (2021) https://doi.org/10.1007/s11845-021–02558-7

The article Early warning scores and critical care transfer – patient heterogeneity, low sensitivity, high mortality, written by Claire C. Nestor, Maria Donnelly, Siobhán Connors, Patricia Morrison and John Boylan, was originally published electronically on the publisher’s internet portal on 10 March 2021 without open access. With the author(s)’ decision to opt for Open Choice, the copyright of the article changed on 31 March 2021 to © The Author(s) 2021 and the article is forthwith distributed under a Creative Commons Attribution 4.0 International License, which permits use, sharing, adaptation, distribution and reproduction in any medium or format, as long as you give appropriate credit to the original author(s) and the source, provide a link to the Creative Commons licence, and indicate if changes were made. The images or other third party material in this article are included in the article’s Creative Commons licence, unless indicated otherwise in a credit line to the material. If material is not included in the article’s Creative Commons licence and your intended use is not permitted by statutory regulation or exceeds the permitted use, you will need to obtain permission directly from the copyright holder. To view a copy of this licence, visit http://creativecommons.org/licenses/by/4.0.

The original article has been corrected.

